# Anti-oxidant effects of cannabidiol relevant to intracerebral hemorrhage

**DOI:** 10.3389/fphar.2023.1247550

**Published:** 2023-09-28

**Authors:** Gaili Yan, Xiangyu Zhang, Hongmin Li, Yan Guo, V. Wee Yong, Mengzhou Xue

**Affiliations:** ^1^ Department of Cerebrovascular Diseases, The Second Affiliated Hospital of Zhengzhou University, Zhengzhou, Henan, China; ^2^ Academy of Medical Science, Zhengzhou University, Zhengzhou, Henan, China; ^3^ Hotchkiss Brain Institute and Department of Clinical Neurosciences, University of Calgary, Calgary, AB, Canada

**Keywords:** intracerebral hemorrhage, oxidative stress, CBD, cannabidiol, Nrf2/ARE (antioxidant response element) pathway

## Abstract

Intracerebral hemorrhage (ICH) is a subtype of stroke with a high mortality rate. Oxidative stress cascades play an important role in brain injury after ICH. Cannabidiol, a major non-psychotropic phytocannabinoids, has drawn increasing interest in recent years as a potential therapeutic intervention for various neuropsychiatric disorders. Here we provide a comprehensive review of the potential therapeutic effects of cannabidiol in countering oxidative stress resulting from ICH. The review elaborates on the various sources of oxidative stress post-ICH, including mitochondrial dysfunction, excitotoxicity, iron toxicity, inflammation, and also highlights cannabidiol’s ability to inhibit ROS/RNS generation from these sources. The article also delves into cannabidiol’s role in promoting ROS/RNS scavenging through the Nrf2/ARE pathway, detailing both extranuclear and intranuclear regulatory mechanisms. Overall, the review underscores cannabidiol’s promising antioxidant effects in the context of ICH and suggests its potential as a therapeutic option.

## 1 Introduction

Worldwide, millions of people die or develop permanent disability because of intracerebral hemorrhage (ICH) (Feigin et al., 2009), and that number is anticipated to rise significantly as the population ages. Currently, clinical trials for ICH treatment mostly focus on surgery, blood and cranial pressure management, and hemostasis (Ren et al., 2020; Schrag and Kirshner, 2020). While these approaches have some control of hematoma size and reduce mortality following ICH, there is insufficient evidence to support the claims that they also improve functional outcomes or quality of life for patients. Therefore, seeking new treatment strategy is essential for enhancing ICH prognosis.

The damage after ICH includes primary and secondary brain injuries (Shao et al., 2019). Primary injury refers to the direct mechanical compression of the hematoma, which produces tissue displacement and disruption. Secondary injury occurs within minutes of ICH and ensues over days to weeks (Xue and Yong, 2020). Depending on damage severity, complex cascades of biochemical events are triggered, such as excitotoxicity by erythrocyte lytic products, inflammation and oxidative stress (OS) (Hu et al., 2016) which influence the overall ICH outcome and prognosis.

OS refers to the imbalance between oxidative and antioxidant systems. Pro-oxidative systems include the overproduction of reactive free radicals, principally reactive oxygen species (ROS) and reactive nitrogen species (RNS). The former comprises superoxide anion radical (•O_2_-), hydroxyl radical (•OH), hydrogen peroxide (H_2_O_2_) and singlet oxygen (^1^O_2_) (Zhang Y. et al., 2022). RNS is mainly composed of nitric oxide (NO), nitrogen dioxide (NO_2_) and peroxynitrite (ONOO^−^). Accumulating evidence suggests that there are multiple sources of ROS/RNS, such as NADPH oxidase (NOX), mitochondria respiratory chain and endothelial nitric oxide synthase (eNOS) (Peoples et al., 2019). Antioxidant systems include non-enzymatic and enzymatic parts. The former mainly consists of lipophilic and hydrophilic antioxidants, such as glutathione and reducing coenzyme Q10. The latter includes superoxide dismutase (SOD), catalase (CAT), glutathione peroxidases (GPXs), peroxiredoxins (PRXs), heme oxygenase-1 (HO-1, HMOX-1) and NAD (P)H dehydrogenase quinone 1 (NQO1) (Cuadrado, 2022).

Many studies have found that ROS/RNS contribute to various physiological processes such as gene expression, protein modification, cell proliferation and differentiation, homeostasis and hypoxia adaptation (Murphy et al., 2011; Zhang B. et al., 2022). However, overproduction of ROS/RNS can occur, attributed to a range of reasons including mitochondrial dysfunction, glutamate excitotoxicity, iron toxicity and pro-inflammatory cells (Zhang Y. et al., 2022). ROS/RNS overabundance causes lipid peroxidation, protein destruction, DNA damage and eventual cell death (Valko et al., 2007).

*Cannabis sativa L*, closely related to marijuana, has been used as an herbal treatment for a variety of diseases for over 1,000 years. It contains hundreds of chemical constituents termed phytocannabinoids with Δ9-tetrahydrocannabinol (THC) and cannabidiol (CBD) as the most abundant (Atakan, 2012). Unlike Δ9-THC which has psychotropic effects, CBD is the major non-psychotropic component and enjoys low abuse potential.

Various studies have employed diverse sources of CBD and different suspension agents for administration, yielding considerable pharmacokinetic variability among formulations (Abbotts et al., 2022). Here we exclusively present the metabolism characteristics of Epidiolex^®^, the only CBD form that has undergone rigorous pharmacokinetic evaluation and earned approval from the U.S. Food and Drug Administration. This specific formulation of CBD demonstrated a dose-dependent, albeit non-linear peak concentration response (Taylor et al., 2018). In healthy adults, the *C*_max_ values for Epidiolex^®^ were 292 ng/mL and 782 ng/mL after 1,500 mg and 6,000 mg doses, respectively (Taylor et al., 2018). At steady state (750 and 1,500 mg CBD twice daily. After 7 days), the time to reach maximal concentration was between 2.5 and 5 h, the distribution volume was between 20,963 and 42,849 L and the protracted elimination half-life was around 60 h (Taylor et al., 2018). Notably, the intake of high-fat/high-calorie meals led to a 4.85-fold increase in CBD plasma exposure (*C*_max_), and a 4.2-fold increase in AUC (area under the curve) (Taylor et al., 2018). CBD is predominantly metabolized within the liver through the involvement of cytochrome P450 (CYP2C19 and CYP3A) as well as uridine 5′-diphosphoglucuronosyltransferase (UGT1A7, UGT1A9, and UGT2B7) (Jiang et al., 2011; White, 2019). Furthermore, CBD may be the inhibitor or inducer of several cytochrome P450 isoforms, which underscores a notable risk for potential drug interactions with CYP substrates. For instance, a pediatric expanded-access study involving 13 participants concurrently using clobazam (a CYP2C19 substrate) and CBD (gradually increased to 20 mg/kg/day) over a 4-week period demonstrated a 60% surge in serum clobazam levels and a 500% increase in the norclobazam metabolite (Wheless et al., 2019). It should also be pointed out that patients with hepatic impairment may necessitate CBD dosage adjustments. In one investigation, AUC levels escalated proportionally with the severity of hepatic impairment, with patients experiencing severe impairment witnessing an approximately 5-fold increase in AUC (Taylor et al., 2019).

In recent years, CBD was reported to be useful for ameliorating symptoms related to epilepsy (Zhan et al., 2022; Reddy et al., 2023), pain (Britch and Craft, 2023), anxiety (Fabris et al., 2022) as well as other neurological diseases (Yang et al., 2022). The antioxidant effects of CBD have been highlighted. The phenolic hydroxyl (-OH) group is a functional group for CBD, and is a good hydrogen donor. Due to the π-electrons delocalization of the benzene ring, the free radicals produced from the -OH group are chemically more stable than those generated from ROS/RNS. Therefore, the reaction of the -OH group and ROS/RNS in a chain reaction can terminate the continued generation of uncontrolled new radicals (Kim et al., 2021). In addition to direct radical-scavenging, the antioxidant effects of CBD are achieved through specific receptor-mediated pathways. There is an endocannabinoid system in the human body that is responsible for pain, sleep, appetite, and immune response (Maccarrone et al., 2023). CBD may also exert antioxidant effects through binding with endocannabinoid receptors CB_1_ and CB_2_. Overall, CBD has better antioxidant capacity than either alpha-tocopherol or ascorbate (Hampson et al., 1998), which indicates that it may be a potential treatment for ICH.

Here, we review the factors that induce OS after ICH, including mitochondrial dysfunction, excitotoxicity, iron toxicity and over-activated inflammatory cells. We discuss the inhibitory effects of CBD on these factors, which contribute to the reduction of ROS/RNS production. The Nrf2/ARE (nuclear factor erythroid-2 related factor 2/antioxidant response element) pathway is a crucial signaling pathway for cellular resistance to OS, as it regulates the expression of multiple proteins that are responsible for free radical elimination. Here, we explore the possible regulatory effects of CBD on key factors in the anti-oxidant pathway, including KEAP1, p62, GSK3β, Nrf2, p65, and BACH1. These effects suggest that CBD could enhance antioxidant capacity including that in ICH. To date, however, few studies have examined CBD in ICH. Thus, the following sections provide instructive effects of CBD which we propose would be relevant for ICH.

## 2 Method

The search was performed in the PubMed database for papers published up to June 2023, using the following search terms: [(intracerebral hemorrhage) AND (oxidative stress)] OR [(cannabidiol) AND (oxidative stress)]. The gathered literature is imported into an Excel table, and after an initial review of the titles, any literature not aligned with the topic is excluded. Following this, the abstracts of the remaining articles are individually assessed, with a focus on closely related articles that pertain to the topic.

## 3 Inhibition of ROS/RNS generation by CBD: relevance for ICH

### 3.1 CBD inhibits ROS/RNS generated from mitochondria dysfunction

ROS/RNS are generated in mitochondria as products of mitochondrial respiration. Approximately 1%-2% of oxygen reacting with electrons leaking from the electron transport chain (ETC) is converted into ROS, especially superoxide anions, during normal physiological respiration (Chance et al., 1979; Zorov et al., 2014). Under conditions of OS, more electrons generated during the citric acid cycle are pushed into the ETC (Brownlee, 2005). At the same time, mitoK_ATP_ is activated and increases K^+^ levels in the mitochondrial matrix which also results in mitochondrial ROS/RNS production from the ETC (Malinska et al., 2010). During ICH, large amounts of ROS/RNS are detected in mitochondria, inducing their disintegration and cell death. A study of 6 brain tissue samples adjacent to the hematoma from patients with ICH showed that mitochondrial respiration declined as early as 2 h following ICH, despite adequate levels of metabolic substrates and O_2_ (Kim-Han et al., 2006).

A significant increase in the production of ROS was detected with MitoSOX Red reagent, an indicator of ROS generated from mitochondria, in HT22 cells suffering oxygen–glucose-deprivation/reperfusion (Sun et al., 2017), in BV-2 cells exposed to lipopolysaccharide (Li M. et al., 2022), and in human coronary artery endothelial cells subjected to high glucose (Rajesh et al., 2007); CBD treatment reduced levels of ROS in these conditions. In models induced by mitochondria-targeted toxins including rotenone (Echeverry et al., 2021), aluminum phosphide (Hooshangi Shayesteh et al., 2022) and oligomycin (Ryan et al., 2009), treatment with CBD restored mitochondrial membrane potential, increase ATP production and thus improved mitochondrial stability and cell viability (Baban et al., 2018; Hooshangi Shayesteh et al., 2022). Taken together, CBD is a potential mitochondria-targeting agent that exerts powerful protective effects under pathological conditions.

The mechanisms that CBD protect mitochondria and reduce mitochondrial ROS production have been reported as follows. CBD significantly increases the expression and activity of the ETC complexes I, II, IV, and V (Sagredo et al., 2007; Ryan et al., 2009; Hao et al., 2015; Hooshangi Shayesteh et al., 2022; Kumar Kalvala et al., 2022) which reduces electron leakage and the overproduction of ROS. In addition, CBD restores energy metabolism. CBD is reported to enhance glucose 6-phosphate dehydrogenase activity and to inhibit abnormal glycolysis and lactate accumulation (Sun et al., 2017; Lu et al., 2021). Cannabidiol also maintains mitochondrial morphology by modulating mitochondrial fission and fusion. (Li M. et al., 2022; Wang et al., 2023; Xu et al., 2023). For example, CBD can inhibit the expression of fission genes including mitochondrial fission 1 protein (FIS1), dynamin-1-like protein (DRP1) and optic atrophy type 1 (OPA1), and increase the expression of fusion genes, such as mitochondrial elongation factor (MIEF1), mitofusin 1 (Mfn1) and mitofusin 2 (Mfn2), *in vitro* model of pulmonary hypertension (Lu et al., 2021).

### 3.2 CBD inhibits ROS/RNS generated from excitotoxicity

Excitotoxicity, a type of glutamate-mediated neurotoxicity, has been a focus of stroke research for the past few decades. Brain tissue contains high concentrations of the excitatory neurotransmitter glutamate, and many neurons contain receptors that respond to glutamate; these make brain tissue susceptible to suffer excitotoxicity (Lai et al., 2014). Following ICH, glutamate release rises while reuptake falls, causing accumulation of excessive glutamate to constantly stimulate the N-methyl-D-aspartate receptor (NMDAR) and induce calcium influx. These lead to intracellular calcium overload and calcium homeostasis imbalance which in turn exacerbates the release of glutamate. Eventually, the calcium-dependent death pathway is activated (Shen et al., 2022). Moreover, high level of intracellular calcium also activates neuronal NOS and NADPH oxidase to synthesize NO and superoxide, respectively (Love, 1999). Consistently, ROS accumulation was detected in brain microvascular endothelial cells treated with glutamate (Parfenova et al., 2006). To sum up, inhibiting excitotoxicity could control the production of ROS/RNS.

ONOO- is a NO derivative and strong oxidant formed by NO and superoxide anion. Excessive formation of ONOO- was elicited in the NMDA-induced rat model of retinal excitotoxicity; administration with CBD reduced ONOO- production and lipid peroxidation, leading to attenuation of retinal neuronal apoptosis and loss (El-Remessy et al., 2003). In traumatic brain injury models, oral CBD pretreatment at all doses (50, 100, or 200 mg/kg) dramatically lessened local glutamate concentration at 30 min post-TBI. Significant suppression of glutamate concentration was also noted at several hours and even weeks (Santiago-Castañeda et al., 2022). In another study, these authors observed that CBD administration lessened glutamate release in synaptosomes collected from cocaine-treated rat hippocampus (Gobira et al., 2015). Additionally, CBD has been demonstrated to lower glutamate levels and excitotoxicity in neonatal animal models of ischemia-hypoxia (Pazos et al., 2012; Pazos et al., 2013; Lafuente et al., 2016).

Although the exact mechanisms by which CBD suppresses the over-release of glutamate and subsequent excitotoxicity are unknown, there are several possibilities. The first likely mechanism involves the endocannabinoid system. The regulatory role of the endogenous cannabinoid system in glutamatergic neurons has been widely reported, particularly for CB_1_ receptors. Although CBD has a low affinity for CB_1_, it can increase CB_1_ agonist endogenous cannabinoid levels by inhibiting cannabinoid hydrolases. Moreover, endogenous cannabinoids system was shown to have neuroprotective effects in excitotoxicity (Kreutz et al., 2009; Grabiec et al., 2012). However, Pazos et al. suggested that both CB_2_ and 5HT_1A_ receptors or their heteromers are involved in anti-excitotoxic effects in an endocannabinoid-independent manner (Pazos et al., 2013). It is also possible that the positive effect of CBD on glutamate level is the result of an increase in brain blood flow brought on by 5HT_1A_ receptor activation, and not a specific neuroprotective effect (Pazos et al., 2013). The sodium-calcium exchanger is an important regulator of excitotoxic calcium homeostasis which extrudes intracellular calcium via driving force of sodium influx (Rodrigues et al., 2022). CBD is proposed to counteract excitotoxicity partly by enhancing the expression of sodium-calcium exchanger (Khaksar and Bigdeli, 2017).

### 3.3 CBD inhibits ROS/RNS generated from iron toxicity

In the late periods of ICH, the erythrocytes from hematoma lyse, releasing hemoglobins. The latter can be phagocytosed by infiltrating microglia or macrophages and metabolized into iron. Then, excess iron will be transported into surrounding neurons (Lu et al., 2022). Iron overload plays a significant role in secondary brain injury as the reaction of iron and H_2_O_2_ via Fenton reaction yields excessive •OH (Wan et al., 2019). A number of studies have demonstrated that iron toxicity causes brain damage after ICH and that reducing iron level with iron chelators attenuates the injury and reduces ROS production (Li et al., 2017; Wang et al., 2021; Zhu et al., 2021).

Few studies have reported on the role of CBD in iron toxicity. Da Silva et al. found abnormal expression of intrinsic apoptotic proteins (Caspase 9, APAF1, Caspase 3, and cleaved PARP) and mitochondrial fusion/fission proteins (DNM1L and OPA1) in iron overload rat models, while treatment with CBD reversed iron-induced damage and recovered proteins expression levels back to values comparable to the control groups (da Silva et al., 2014; da Silva et al., 2018b). They also found that CBD rescued the reduced levels of methylcytosine and hydroxymethylcytosine in mitochondrial DNA induced by iron overdose (da Silva et al., 2018a).

Interestingly, because phenolic and polyphenolic compounds has iron binding affinity (Horniblow et al., 2017; Kejík et al., 2021), Antonyová et al. reported that CBD stably binds ferrous iron as tested by UV-Vis spectroscopy; it acted as a chelator and strongly inhibited (IC_50_ = 4.8 μM) a Fe(II)-dependent protein, ten-eleven translocation methylcytosine dioxygenase 1, which converts 5-methylcytosine to 5- hydroxymethylcytosine (Antonyová et al., 2022). Given its dual action of iron chelation and inhibition of heme oxygenase mentioned in the next part, CBD holds promise as a treatment for iron toxicity or ferroptosis.

### 3.4 CBD inhibits ROS/RNS generated from inflammatory cells

Pathological analysis of ICH has revealed that resident microglia are activated by blood-derived products within 1 hour after stroke onset. Subsequently, other immune cells in the blood, especially neutrophils, also enter the brain and migrate around the haematoma (Xue and Yong, 2020). In addition to releasing inflammatory factors, these immune cells that are initially recruited for debris clearance are also involved in ROS/RNS production.

CBD was reported to reduce NO and ROS production, induced by lipopolysaccharide, in BV-22 cells and primary microglia. (Sonego et al., 2018; Dos-Santos-Pereira et al., 2020; Kim et al., 2021). ROS production was decreased with 1 μM CBD by over 70% and returned to control levels with 10 μM (Dos-Santos-Pereira et al., 2020). The inhibitory effect of CBD on microglia activation, as indicated by Iba-1, is observed in various experimental models *in vitro* and vivo (Kozela et al., 2011; Ceprián et al., 2017; Meyer et al., 2022). For example, CBD decreased the release of proinflammatory mediators (TNF-α and IL-1β) and chemotactic factors from microglia (Kozela et al., 2010; Dos-Santos-Pereira et al., 2020). Interestingly, some of the findings showed that CBD enhanced phagocytosis capability of microglia while inhibiting their activation. In organotypic oxygen-glucose deprivation (OGD) slices, CBD treatment was accompanied by fewer activated microglia but more rod microglia, which tended to migrate to the damaged area (Lana et al., 2022). In summary, on the one hand, CBD can suppress inflammatory events associated with excessive activation of microglia; on the other hand, it promotes hematoma clearance by microglia, both of which are beneficial in reducing ROS/RNS production and improving ICH prognosis.

The transient receptor potential (TRP) channel, particularly the TRPV2 channel, appears to be the most likely receptor involved in the phagocytosis effects mentioned above (Yang et al., 2022). TRPV2, located mainly in neurons, decreased significantly in OGD (Lana et al., 2022) and Alzheimer’s disease models (Yang et al., 2022). Nevertheless, CBD enhances its translocation to microglia in injury models, predominantly in the membrane fraction. Moreover, CBD enhancement of microglia phagocytosis was blocked by TRPV2 knockdown (Yang et al., 2022) or by a TRP channel blocker, ruthenium red (Hassan et al., 2014).

**FIGURE 1:**
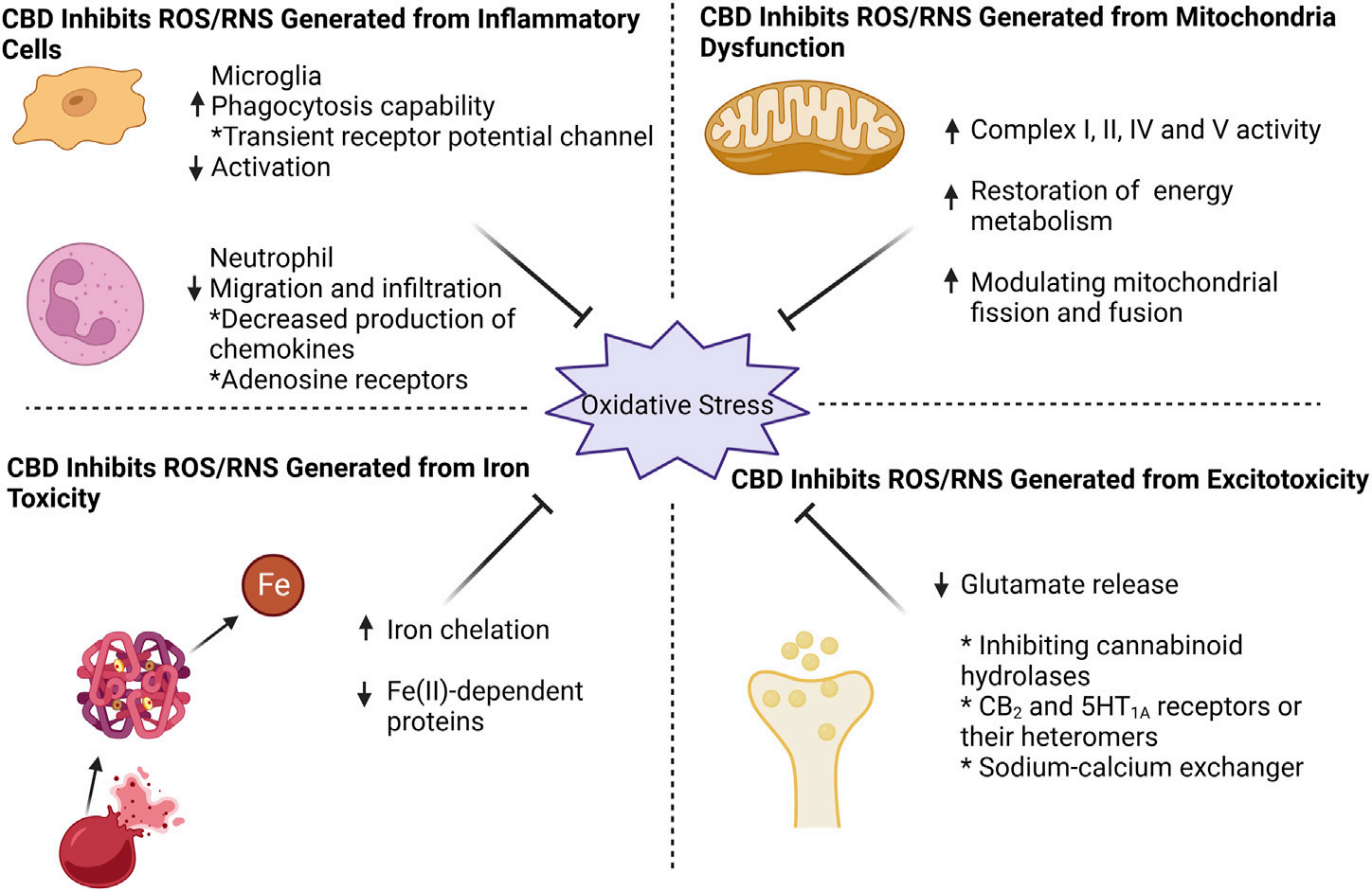
CBD effectively counteracts oxidative stress by targeting four key aspects: mitochondrial dysfunction, excitotoxicity, iron toxicity, and inflammatory cells. CBD enhances mitochondrial respiratory chain complex activity, restores mitochondrial energy metabolism, and modulates mitochondrial fission and fusion. For excitotoxicity, CBD inhibits glutamate release, probably by (i) inhibition of cannabinoid hydrolases activity (ii) direct action on CB_2_ and 5HT_1A_ receptors or their heteromers, and (iii) action on sodium-calcium exchangers to reduce calcium inward flow. CBD can chelate iron ions and thus exhibit an inhibitory effect on iron-dependent proteins. When it comes to inflammatory cells, CBD effectively suppresses microglia activation while enhancing their phagocytic capacity through transient receptor potential channel. Additionally, CBD reduces neutrophil infiltration, potentially by targeting chemokine and adenosine receptors.

Previous literature shows that CBD inhibits neutrophil migration and infiltration *in vivo* and vitro (Mabou Tagne et al., 2019; Gómez et al., 2021; Robaina Cabrera et al., 2021). Thus, treatment with CBD may ameliorate excessive inflammatory responses involving neutrophils. This may be attributed to the inhibition of chemokine production by CBD (Wang et al., 2017). Some work suggested that CBD inhibits neutrophil recruitment via adenosine receptors A_2A_ (Ribeiro et al., 2012) or 5-HT_1A_ (Thapa et al., 2018), but strong evidence is lacking. Moreover, CBD reduces ROS production directly from neutrophils induced by fMLP (Mabou Tagne et al., 2019) or isolated from mice and patients with chronic binge alcohol diet (Wang et al., 2017) (Figure 1; Table 1).

**TABLE 1:** Mechanism of cannabidiol inhibiting ROS/RNS production.

References	CBD dosing	Results
Hao et al. (2015)	Male C57BL/6J mice, 10 mg/kg, i.p., once daily for 5 days	Improving mitochondrial complex I, II activity
Hooshangi Shayesteh et al. (2022)	Adult male Albino Wistar rats, 5, 25, 50, and 100 μg/kg, i.v for 12 and 24 h	Improving mitochondrial complex I, IV activity
Kumar Kalvala et al. (2022)	Primary dorsal root ganglions neuronal cells, 12 µM	Improving mitochondrial complex I activity
Sagredo et al. (2007)	Adult male Sprague Dawley rats, 5 mg⁄ kg, i.p. once daily for 5 days	Reducing the striatal atrophy caused by inhibitor of mitochondrial complex II
Ryan et al. (2009)	Human neuroblastoma cell line, 1 μM	Increasing cell viability insulted by complex V inhibitor, oligomycin, and uncoupler of ATP synthesis, FCCP
Sun et al. (2017)	HT22 cells, 5 μM	Increasing cells’ ATP production-linked OCR insulted by complex V inhibitor, oligomycin, Increasing cells’ maximal respiration and the spare respiratory capacity insulted by uncoupler of ATP synthesis, FCCP
Lu et al. (2021)	Male C57BL/6J mice, 10 mg/kg i.g. once daily for 12 days	Inhibiting the expression of fission genes including FIS1, DRP1 and OPA1; Increasing the expression of fusion genes including MIEF1, Mfn1 and Mfn2
Li et al. (2022b)	BV2 microglia cell line, primary microglia, 1 μM, C57BL/6 J mice, 10 mg/kg, i.p., once daily	Suppressing microglia activation, Increasing fusion genes Mfn2 expression
Wang et al. (2023)	Male C57BL/6J mice, 10 mg/kg, i.g., once daily for 30 days	Increasing the expression of Mfn1, Mfn2, Opa1; Decreasing the expression of Drp1
Grabiec et al. (2012)	Organotypic hippocampal slice, 100p.m.-10 μM	Inhibiting excitotoxicity, Reducing the number of positive degenerative cells
Pazos et al. (2013)	Newborn pigs, 1 mg/kg, single dose	Decreasing glutamate/N-acetylaspartate ratio partly via 5HT_(1A)_ and CB_2_ receptors
Khaksar and Bigdeli (2017)	Adult male Wistar rats, 50, 100, and 200 ng/rat; i.c.v. for 5 days	Increasing NCX2 and NCX3 expression
Antonyová et al. (2022)	-	Chelating iron ions. Inhibiting iron-dependent proteins
Yang et al. (2022)	Primary microglia, 5 μM	Enhancing microglial Aβ phagocytosis via the TRPV2 channels
Hassan et al. (2014)	BV-2 microglia, 10 μM	Enhanceing microglial phagocytosis via TRP channel
Wang et al. (2017)	C57BL/6 J mice, 5 or 10 mg/kg/day, i.p., 11 days	Reducing neutrophil infiltration and chemokines production
Ribeiro et al. (2012)	Male C57BL/6 mice 20 mg/kg, i.p., single dose	Reducing neutrophil infiltration and chemokines production possibly by adenosine A(2A) receptor
Thapa et al. (2018)	Male BALB/c mice 5 μL 5% CBD, topical	Reducing neutrophil infiltration possibly by 5-HT_1A_ receptors

DRP1, dynamin-1-like protein; FCCP, carbonyl cyanide-p-trifluoromethoxyphenyl hydrazone; FIS1, mitochondrial fission 1 protein; i.c.v., intracerebroventricular; i.g., intragastric; i.p. intraperitoneal; i.v. intravenous; Mfn1, mitofusin 1; Mfn2, Mitofusin 2; MIEF1, mitochondrial elongation factor; NCX, sodium-calcium exchanger; OCR, oxygen consumption rate; OPA1, optic atrophy type 1; TRP, transient receptor potential.

## 4 CBD promotes the scavenging of ROS/RNS: relevance for ICH

There are a variety of defense mechanisms, including detoxification and antioxidant enzymes, to counteract the over-production of free radicals after ICH. Many of these enzymes are under the control of the Nrf2/ARE pathway which is currently regarded as the major system in cellular antioxidant response (Zhao et al., 2007; Loan et al., 2022). Nrf2 was reported to increase significantly at 2 h, peaking at 24 h in the perihematomal region in rats with ICH (Shang et al., 2013). Furthermore, Nrf2 knockdown mice underwent more serious brain damage and Nrf2 activation reduces peroxide formation (Zeng et al., 2017). Therefore, activation of Nrf2 by drugs is a promising target for attenuating OS-induced brain damage after ICH.

**FIGURE 2:**
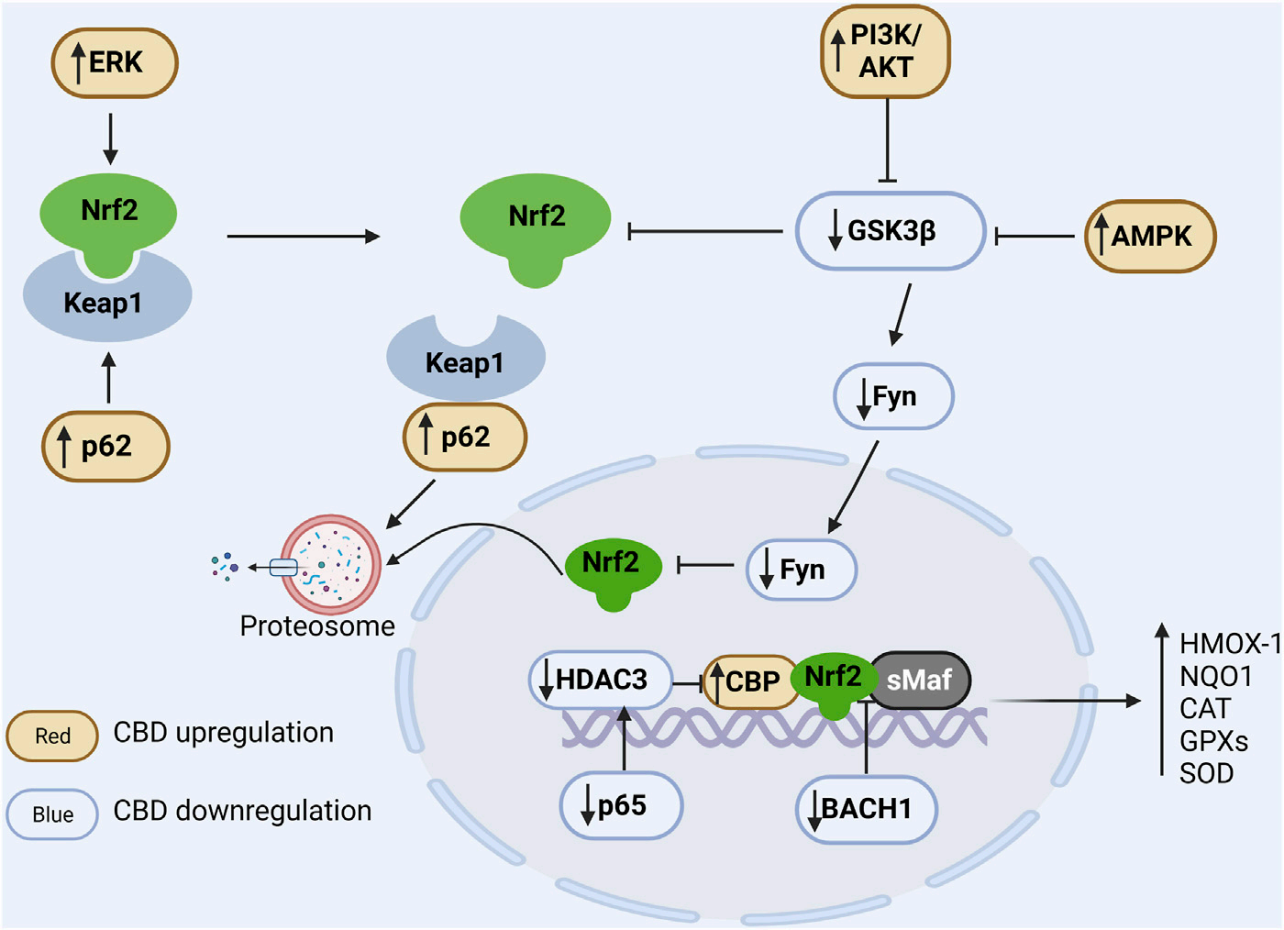
Regulation of CBD on Nrf2/ARE pathway. In the cytoplasm, Nrf2 is ubiquitinated by Keap1 and degraded by the proteasome under physiological conditions. Positive regulation of ERK and p62 by CBD via phosphorylation of Nrf2 and binding competitively of Keap1, respectively, promotes dissociation of Nrf2 from Keap1. CBD downregulates GSK3β to favor Nrf2 accumulation, and it can further enhance GSK inhibition by upregulating PIK and AMPK. In the nucleus, Fyn phosphorylated by GSK promotes the nuclear export of Nrf2 and CBD can downregulate Fyn expression. Additionally, BACH1 can competitively bind sMaf and ARE and thus inhibit the expression of antioxidant genes, while CBD can inhibit the competitive binding of BACH1 to sMaf and ARE. Moreover, CBD reduces the expression of the NFκB subunit p65 to suppress its induced deacetylation environment. In conclusion, CBD can modulate the antioxidant pathways through multiple ways, resulting in a significant increase in antioxidant gene expression.

Under physiological conditions, Nrf2 is negatively regulated by Keap1 and maintained at a low level. In detail, Keap1 homodimerizes and binds to an E3 ubiquitin ligase complex via cullin-3 (Keap1-Cul3-RBX1 complex), which mediates Nrf2 ubiquitination and subsequent 26S proteasome degradation. Under stress conditions, like excessive ROS or electrophiles, the Keap1-Nrf2 binding is impaired, leading to Nrf2 stabilization and nuclear translocation (Buendia et al., 2016; Fão et al., 2019). In the nucleus, Nrf2 binds to small musculoaponeurotic fibrosarcoma (sMaf) proteins and the created complex identifies specific antioxidant response elements (AREs), promoting the transcription of genes encoding free radical scavenging proteins, including HMOX-1, NQO1, CAT, GPXs, and SOD. Here the regulation of the Nrf2/ARE pathway by CBD is described as extranuclear and intranuclear regulation, and the former mechanism can be further divided into Keap1-dependent and Keap1-independent pathways (Figure 2).

### 4.1 Extranuclear regulation

#### 4.1.1 Keap1-dependent pathway

In the environment of OS, the Keap1 cysteine residues are modified resulting in changes in its conformational and consequently Nrf2 release (Kopacz et al., 2020). In human oral keratinocytes induced by 5-fluorouracil, CBD (0.5, 2.5, and 5 μM) promotes Keap1 degradation and Nrf2 stabilization, which in turn enhances Nrf2 target genes expression and cellular antioxidant capacity (Li L. et al., 2022). In addition, p62, a polyubiquitination binding protein, competes for the binding site of Nrf2 to Keap1, resulting in Keap1 autophagy degradation and Nrf2 activation (Komatsu et al., 2010). It has been observed that CBD increases the level of p62 in primary human keratinocytes (Jastrząb et al., 2019) and in rats with eccentric contractions (Langer et al., 2021).

#### 4.1.2 Keap1-independent pathway

There are several proteins that regulate the Nrf2/ARE pathway mainly through the phosphorylation of Nrf2. Indeed, Nrf2 contains several phosphorylation sites that have been described as crucial regulators of Nrf2 activation and degradation. For example, Glycogen synthase kinase-3β (GSK-3β) induces ubiquitinated degradation of Nrf2 via phosphorylation in the cytosol (Saha et al., 2020). CBD administration *in vitro* suppressed GSK3β activation in PC12 neuronal cells (Esposito et al., 2006) favoring Nrf2 stabilization. It is known that both PI3K/Akt and AMPK pathways can indirectly mediate Nrf2, partly by inhibiting GSK-3β activity (Deng et al., 2019; Lv et al., 2019). An upregulation of PI3k/Akt and AMPK has been reported after treatment with CBD. At 5 μM concentration, CBD was reported to inhibit GSK-3β activity likely by promoting the PI3K/Akt pathway in mesenchymal stem cells (Libro et al., 2016). The combination of CBD and tetrahydrocannabinol reversed the decrease in AMPK induced by paclitaxel (Kumar Kalvala et al., 2022). Several proteins, including protein kinase C (PKC), casein kinase II (CK2), and endoplasmic reticulum kinase (ERK) can also phosphorylate Nrf2, leading to its dissociation from Keap1 and nuclear accumulation. Of these, only ERK has been shown to be positively regulated by CBD (Alegre-Zurano et al., 2022).

### 4.2 Intranuclear regulation

BTB And CNC Homology 1 (BACH1) competes with Nrf2 for binding sMaf to form BACH1-sMaf heterodimer which also recognizes AREs. Unlike the Nrf2-sMaf complexes, the BACH1-sMaf primarily acts as a transcriptional repressor (Oyake et al., 1996; Liu et al., 2022). Previous literature has indicated that BACH1-sMaf represses NQO1 and HMOX-1 gene expression in a variety of cell lines (Sun et al., 2002; Dhakshinamoorthy et al., 2005; Reichard et al., 2007). Using siRNA to silence BACH1, HMOX-1 expression is strongly upregulated in HaCaT cells and Huh-7 hepatocytes (Shan et al., 2004; Casares et al., 2020). Importantly, treatment with CBD induces HMOX-1 expression in HaCaT cells (mean 58-fold), but its function is greatly impaired in BACH1 knocked-down cells (mean 10-fold). In other words, CBD can promote HMOX1 expression by facilitating the nuclear export and degradation of BACH1 (Casares et al., 2020). In addition, Nrf2 transcription activity is also negatively mediated by p65 (RelA), a subunit of NF-κB complex. Liu and others found that p65 selectively deprives CBP (CREB binding protein, a major coactivator of Nrf2) of Nrf2 and enhances the recruitment of histone deacetylase 3 (HDAC3) which deacetylates CBP and thus suppresses CBP coactivator activity, resulting in local histone hypoacetylation (Liu et al., 2008). The inhibitory effect of CBD on p65 has been reported elsewhere. CBD administration inhibits p65 phosphorylation and subsequent nuclear translocation, thereby suppressing the expression of its target genes such as tumor necrosis factor (TNF) (Huang et al., 2019; Lubschinski et al., 2022). Nrf2 also can be phosphorylated by Fyn kinase, a member of Src family member, leading to Nrf2 nuclear export and degradation (Jain and Jaiswal, 2007; Kaspar and Jaiswal, 2011). Downregulation of Fyn was observed in peripheral blood mononuclear cells collected from patients with multiple sclerosis treated with CBD for 4 weeks (Sorosina et al., 2018). Moreover, Fyn protein has also been shown to be phosphorylated by GSK3β mentioned above, which causes its nuclear transport (Demuro et al., 2021). Overall, CBD inhibits Nrf2 repressor BACH1, p65 and Fyn favoring Nrf2 activity. Of these, CBD appears to have the most pronounced effect on BACH1.

In summary, given the protective role of the Nrf2/ARE pathway after ICH mentioned above and the potent activation of this pathway by CBD, we can infer that CBD may significantly activate the Nrf2/ARE pathway after ICH, thereby promoting expression of multiple antioxidant gene and enhancing antioxidant capacity.

In addition to the anti-oxidant effects covered in the current review, Henry and others, in their review, suggest that CBD may also have exciting anti-inflammatory, vascular effects, and neuroprotective function in subarachnoid hemorrhage (Henry et al., 2023). We believe that these may also be potential protective mechanisms for ICH. In other words, CBD may protect against ICH from different ways.

While this review and previous studies propose the potential anti-inflammatory and anti-oxidant effects of CBD against ICH, it is important to note that research in this area is still in its initial stage. There remain numerous unexplored avenues that warrant further investigation. Previous preclinical studies utilize a diverse range of vehicles and doses for CBD administration, resulting in considerable variability in bioavailability. This variability poses challenges in effectively comparing findings across different studies. Thus, a more comprehensive understanding of the pharmacokinetics derived from preclinical research could substantially enhance future CBD investigations. Furthermore, the commonly employed preclinical models for ICH encompass the autologous blood injection model and the collagenase model. Each model boasts distinct characteristics, and evaluating the effectiveness of cannabidiol on both models could offer valuable theoretical insights for subsequent clinical trials.

Moreover, with the increased interest in cannabinoids, more and more clinical trials related to CBD are being conducted. Alarmingly, however, some of these trials have been conducted without adequate theoretical support and rigorous experimental design. We suggest that preclinical studies of CBD in ICH or other aged-related degenerative conditions should be conducted to fully assess the actual effects of CBD, and then clinical trials could be carried out if necessary.

## 5 Conclusion

The review summarizes the inhibition of ROS/RNS production and the promotion of ROS/RNS elimination by CBD. These results suggest that CBD may be an effective treatment against ICH-induced OS. However, several recent reports have also indicated that CBD can promote ROS production in cancer cells to induce cell apoptosis (Yan et al., 2023). The conflicting results may be partly related to the type of cells examined. Therefore, it is necessary to conduct comparative studies with various cell types. Furthermore, previous work on the anti-oxidant effects of CBD seems to be attributed more to its -OH group, when in fact CBD has been identified as a ligand for several receptors, especially the endogenous cannabinoid receptor. The further work on its related receptors in relation to antioxidant effects is necessary. Moreover, it seems imperative to test CBD first in preclinical models of ICH and then in patients with ICH.

## Abbreviations

ARE, Antioxidant response element; BACH1, BTB And CNC Homology 1; CAT, catalase; CBD, Cannabidiol; CBP, CREB binding protein; ETC., electron transport chain; GPXs, glutathione peroxidases; GSK-3β, Glycogen synthase kinase-3β; HO-1 or HMOX-1, heme oxygenase-1; ICH, Intracerebral hemorrhage; NCX, sodium-calcium exchanger; NQO1, NAD (P)H dehydrogenase quinone; Nrf2, nuclear factor erythroid-2 related factor 2; OS, oxidative stress; OGD, oxygen-glucose deprivation; -OH, phenolic hydroxyl; ONOO^−^, peroxynitrite; RNS, reactive nitrogen species; ROS, reactive oxygen species; sMaf, small musculoaponeurotic fibrosarcoma; SOD, superoxide dismutase; THC, Tetrahydrocannabinol; TRP, transient receptor potential.
